# Reproductive Isolation Among Three Nocturnal Moth-Pollinated Sympatric *Habenaria* Species (Orchidaceae)

**DOI:** 10.3389/fpls.2022.908852

**Published:** 2022-06-22

**Authors:** Hai-Ping Zhang, Zhi-Bin Tao, Judith Trunschke, Mani Shrestha, Daniela Scaccabarozzi, Hong Wang, Zong-Xin Ren

**Affiliations:** ^1^CAS Key Laboratory for Plant Diversity and Biogeography of East Asia, Kunming Institute of Botany, Chinese Academy of Sciences (CAS), Kunming, China; ^2^University of Chinese Academy of Sciences, Beijing, China; ^3^CAS Key Laboratory of Aquatic Botany and Watershed Ecology, Wuhan Botanical Garden, Chinese Academy of Sciences (CAS), Wuhan, China; ^4^Department of Disturbance Ecology, Bayreuth Center of Ecology and Environmental Research (BayCEER), University of Bayreuth, Bayreuth, Germany; ^5^School of Pharmaceutical Science and Technology, Tianjin University, Tianjin, China; ^6^School of Molecular and Life Sciences, Curtin University, Bentley, WA, Australia; ^7^Lijiang Forest Biodiversity National Observation and Research Station, Lijiang, China

**Keywords:** hawkmoth, orchid, pollination, co-existence, pre- and post-pollination barriers

## Abstract

Comparison and quantification of multiple pre- and post-pollination barriers to interspecific hybridization are important to understand the factors promoting reproductive isolation. Such isolating factors have been studied recently in many flowering plant species which seek after the general roles and relative strengths of different pre- and post-pollination barriers. In this study, we quantified six isolating factors (ecogeographic isolation, phenological isolation, pollinator isolation, pollinia-pistil interactions, fruit production, and seed development) that could possibly be acting as reproductive barriers at different stages among three sympatric *Habenaria* species (*H. limprichtii*, *H. davidii*, and *H. delavayi*). These three species overlap geographically but occupy different microhabitats varying in soil water content. They were isolated through pollinator interactions both ethologically (pollinator preference) and mechanically (pollinia attachment site), but to a variable degree for different species pairs. Interspecific crosses between *H. limprichtii* and *H. davidii* result in high fruit set, and embryo development suggested weak post-pollination barriers, whereas bidirectional crosses of *H. delavayi* with either of the other two species fail to produce fruits. Our results revealed that pollinators were the most important isolating barrier including both ethological and mechanical mechanisms, to maintain the boundaries among these three sympatric *Habenaria* species. Our study also highlights the importance of a combination of pre-and post-pollination barriers for species co-existence in Orchidaceae.

## Introduction

Mechanisms of reproductive isolation (RI), preventing the production or survival of hybrid offspring from different species, are the primary criterion for defining species in multicellular, sexual organisms (Barraclough, 2019; Fachardo and Sigrist, 2020). Reproductive barriers result in speciation within a lineage, determining the extent of biodiversity in communities (Lowry et al., 2008; Sweigart and Willis, 2012; Butlin and Smadja, 2018; Volis et al., 2021). While reports of reproductive isolation among angiosperm lineages are common, relatively few studies compared and quantified the relative strengths of different reproductive isolation barriers (Ramsey et al., 2003; Rieseberg et al., 2006; Martin and Willis, 2007; Lowry et al., 2008; Sobel et al., 2010). To do that, it is required to dissect individual components of isolation among distinct species pairs and the relative contribution of each barrier to total isolation (Coyne and Orr, 2004; Nosil et al., 2005; Cozzolino and Scopece, 2008; Widmer et al., 2009).

In plants, mechanisms promoting reproductive isolation can be broadly classified into pre- and post-pollination barriers (Grant, 1994). Pre-pollination barriers include (i) ecogeographic (spatial) isolation, (ii) flowering phenology, and (iii) pollinator isolation including mechanical isolation and ethological barriers (i.e., behavioral preference) that reduce or prevent interspecific mating (Grant, 1994; Dellòlivo et al., 2011; Paudel et al., 2018). In contrast, post-pollination barriers may either impede pollen germination and pollen tube growth in the pistils after pollen is deposited on the stigma or reduce hybrid fitness (Dellòlivo et al., 2011; Paudel et al., 2018; Munguia-Rosas and Jacome-Flores, 2020), and can include pollen-pistil recognition and rejection, pollen tube competition, failure to form hybrid seeds, and inviability of hybrids.

In orchids, reproductive isolation due to the interactions with pollinators has been generally thought to be strong, because some orchids have specialized pollination mechanisms (Cozzolino and Widmer, 2005a; Schiestl and Schlüter, 2009). Floral isolation may be mediated by interspecific differences in floral coloration, floral scent, nectar rewards, and functional morphology. Differences in floral phenotype or chemotype may result in either ethological isolation if pollinators show strong preferences toward one or the other morph, or mechanical isolation, if pollinator cross-visitation does not lead to efficient pollen transfer (Levin, 1971; Grant, 1994; Rieseberg and Willis, 2007). For example, among different orchid species that share the same primary pollinators, interspecific isolation may occur mechanically as the same pollinator receives pollinia at different parts of its body (Nilsson, 1983; Maad and Nilsson, 2004; Schiestl and Schlüter, 2009).

Post-pollination mechanisms of isolation are, by contrast, usually assumed to be weak or less important in orchids (Cozzolino et al., 2004, 2005; Cozzolino and Widmer, 2005a,b). However, in some cases where pre-pollination barriers were weak, post-pollination barriers may play a key role in preventing the formation of hybrids (Cozzolino et al., 2004, 2005; Cozzolino and Widmer, 2005a,b; Sun et al., 2011; Zhang and Gao, 2017). For example, based on molecular, cytogenetic, and morphological analyses and interspecific hand-crosses, Pinheiro et al. (2015) found that strong postzygotic isolation prevents introgression between two hybridizing Neotropical orchids, *Epidendrum denticulatum* and *E. fulgens*. Therefore, it is important to quantify multiple pre- and post-pollination reproductive isolation barriers among related species and their relative contribution to total reproductive isolation.

*Habenaria* Willd., is the largest, terrestrial orchid genus with approximately 800 described species (Pridgeon et al., 2001). It is distributed widely from temperate to tropical regions, although Brazil, southern and central Africa, and eastern Asia are the centers of diversity (Kurzweil and Weber, 1992; Batista et al., 2006, 2013). In many regions, *Habenaria* species with different genetic alliances occur sympatric and with overlapping flowering periods from summer to autumn (Pedron et al., 2012; Zhang and Gao, 2021). Therefore, *Habenaria* spp. are ideal for evaluating the importance of pre- *versus* post-pollination barriers since much is known about the pollination biology and breeding systems in this genus from previous studies mainly focusing on single species (Singer et al., 2007; Peter et al., 2009; Tao et al., 2018a; Xiong et al., 2019; Chen et al., 2021), but the mechanisms allowing the maintenance of their co-existence are not well understood. Only a few studies have documented interspecific isolation mechanisms, and none quantified the relative contribution of pre- or post-pollination factors in this species-rich orchid genus (Zhang and Gao, 2017, 2021).

In this study, we focused on three sympatric congeners in the genus *Habenaria*, *H. limprichtii* Schltr., *H. davidii* Franch. and *H. delavayi* Finet. with different phylogenetic distances between each other to quantify multiple pre- and post-pollination reproductive isolation barriers. Recent phylogenetic analysis showed that *H. davidii* and *H. limprichtii* are sister species within superclade VI, they have a distant relationship with *H. delavayi*, which is not in the same clade (Jin et al., 2017). Scopece et al. (2010) suggested that the strength of post-pollination isolation among species in orchids was distantly related to their phylogenetic closeness (i.e., within the same genus or different genera). Therefore, such co-blooming sympatric congeners in *Habenaria* provide an opportunity to investigate and test the hypothesis for reproductive isolation between species with a different distance of phylogenetic relation to see whether post-pollination isolation mechanisms have evolved.

We identified and measured six different reproductive barriers that are potentially involved in the maintenance of species boundaries among them. We used a combination of approaches including field observations and experiments to document potential factors influencing the evolutionary trajectories between the three species pairs by addressing the following questions: (1) To what extent do the geographic ranges and habitat preferences (quantified by the soil moisture content) overlap between the three species? (2) Do the species differ in floral morphology as well as pollinator communities? (3) What is the extent of the following potential isolation factors influencing the evolutionary trajectories between the three species pairs: (i) phenological isolation, (ii) pollinator isolation (pollinator fidelity and pollinaria deposition on the insect’s body), (iii) pollinia-pistil interactions (interspecific pollen tube growth), (iv) interspecific fruit set rates and (v) embryonic development following interspecific hand-pollinations? (4) What is the relative contribution of pre- *versus* post-pollination barriers to the maintenance of species integrity? Our assessment of the generality of reproductive isolation barriers among these sympatric species will provide new insights for a better mechanistic understanding of speciation and diversification in Orchidaceae.

## Materials and Methods

### Study Species and Sites

**FIGURE 1:**
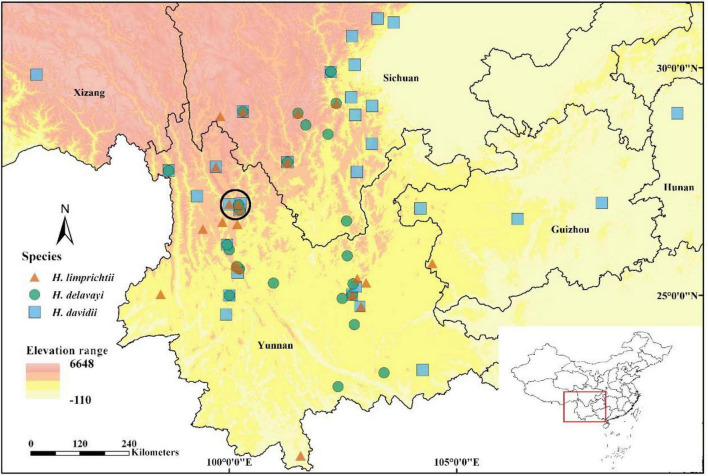
Geographic ranges of three *Habenaria* species based on localities from herbarium collections, GBIF, and our own field survey from 2017 to 2021. The circled area (black circle) comprises the population sites used in our study in the surrounding of the city of Lijiang, northwestern Yunnan.

**FIGURE 2:**
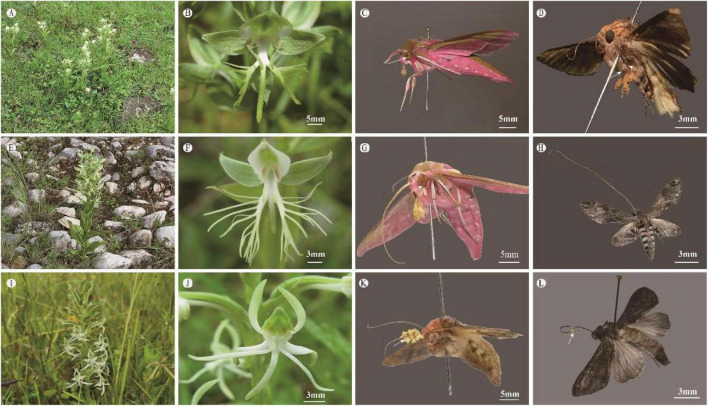
The floral morphology and pollinators of three *Habenaria* species at Yulong Snow Mountain, Lijiang, southwestern Yunnan, China. The habitat **(A)** and flowers **(B)** of *H. limprichtii*; **(C)** pinned specimen of *Deilephila elpenor* subsp. *lewisii* with pollinaria of *H. limprichtii* deposited on its eyes; **(D)** pinned specimen of *Trichoplusia intermixta* with pollinaria of *H. limprichtii* deposited on the lateral-ventral side of the thorax and the femora of middle and hind-legs; the habitat **(E)** and flowers **(F)** of *H. davidii*; **(G)** pinned specimen of *Deilephila elpenor* subsp. *lewisii* with pollinaria of *H. davidii* deposited on its eyes; **(H)** pinned specimen of *Agrius convolvuli* with long proboscis and without carrying any pollinaria of *H. davidii*; the habitat **(I)** and flowers **(J)** of *H. delavayi*; **(K)** pinned specimen of *Trichoplusia intermixta* with pollinaria of *H. delavayi* deposited on proboscis; **(L)** pinned specimen of *Apamea* sp. with one pollinarium of *H. delavayi* deposited on the proboscis.

The three species, *H. limprichtii, H. davidii* and *H. delavayi*, are terrestrial orchids that occur in the mountains of Southwest China, predominantly in the Yunnan and Sichuan provinces (Figure 1). Across their distribution ranges from 800 m a.s.l. up to 3,500 m a.s.l, these three *Habenaria* species are found in diverse habitats, from open grassland to evergreen forest and open grassy pine forest, even steeply sloping rock faces or crevices in rock faces. However, *H. limprichtii* and *H. delavayi* are more likely in the grassy meadows (Figures 2A,I), while *H. davidii* is in a dry and rocky environment (Figure 2E). At the time of flowering, all three species produce a single inflorescence comprising multiple acropetal opening flowers. All three species are exclusively visited by pollinators at nighttime, and individual flowers are consistent with a nocturnal pollination syndrome (Tao et al., 2018a). Flowers of all three species have white or greenish-white petals as perceived by humans (Figures 2B,F,J), produce nectar in floral spurs, and emit a strong aromatic scent after sunset, which can be distinguished among species by humans. Based on the cytological study by Luo (2004), *H. delavayi* is diploid with 2n = 42.

We conducted field experiments in Lijiang, southwestern Yunnan, China, at six study sites from the mid of July (flowering) to the end of October (fruiting) in 2016 and 2017 (Supplementary Figure 1; Tao et al., 2018a for site information of *H. limprichtii*). At our sites in Lijiang, *H. limprichtii* is growing in wet, humid meadows with high-water content, *H. davidii* is growing in dry grasslands and forests with rocky calcareous soils, while *H. delavayi* is growing in grasslands, intermediate between these two extremes.

### Pre-pollination Barriers

#### Geographic Range and Habitat Differentiation

We compiled herbarium data (Chinese Virtual Herbarium), GBIF (data of China from 1000 to 2021), and our own data (Supplementary Table 1 for all data sources) to produce a range map in ArcGIS. We excluded all repetitive samples, misidentified specimens (the number of misidentified specimens accounting for 5.2% for *H. davidii* and 9.2% for *H. delavayi*), and those without GPS coordinates. By including our own data, 87 specimens of *H. limprichtii* (*n* = 24), *H. davidii* (*n* = 34), and *H. delavayi* (*n* = 29) were included to map species distribution (Figure 1). We treated any of the two species co-recorded within a 1 × 1 longitude/latitude grid without elevation difference as sympatric distribution.

**FIGURE 3:**
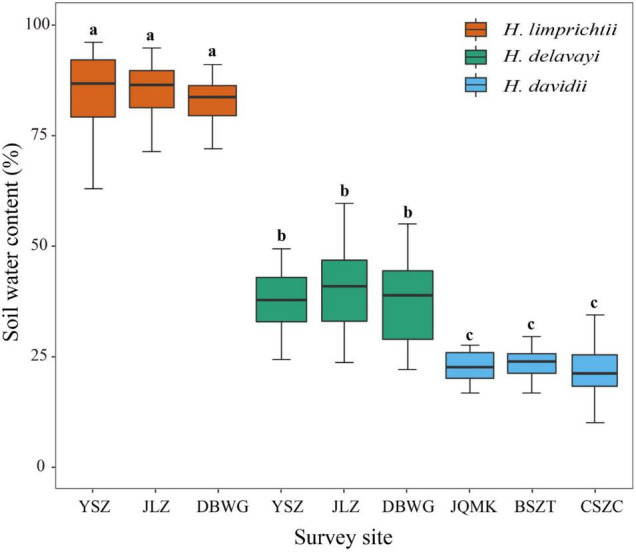
Comparison of soil water content at different sites among three *Habenaria* species at Yulong Snow Mountain, Lijiang. Lowercase letters indicate significant differences at *p* < 0.01.

To quantify differences in microhabitat conditions, we measured soil water content at three sites for each of the three *Habenaria* species (*H. limprichtii* and *H. delavayi* share sites but occur in different microhabitats; Figure 3) following the method used in Belluau and Shipley (2018). We used a soil moisture meter (model: CM-WSY, Hengmei Tech. Inc., Shangdong, China) to do the measurement in the field. To minimize the impact of rainfall on soil moisture, we measured the soil’s water content two days following a rainstorm and calibrated the equipment each time when we measured the soil moisture content. We measured the soil moisture content at the same soil depth of orchids’ tubers, which was 4 cm for *H. limprichtii* (*n* = 101) and *H. davidii* (*n* = 115) and 2 cm for *H. delavayi* (*n* = 108). To avoid damage to the roots and subterranean organs, each probe was inserted 2–3 cm away from the scape of each plant.

#### Floral Phenology and Temporal Isolation

We recorded the flowering phenology at each site as the period between the day when the first flower was noted open on an inflorescence and the day when all the perianth segments on all the flowers in that site had turned black. Observations in each site were carried out at a 4–6-day interval. All flowering individuals of *H. limprichtii* (*n* = 30), *H. davidii* (*n* = 27), and *H. delavayi* (*n* = 34) in each site were observed and recorded for their flowering phenology.

We calculated the strength of phenological asynchrony as an isolating barrier following the equation 4C in Sobel and Chen (2014):


(1) RIpheno=1-(S/(S+U))

where S refers to the proportion of flowering overlap time between any two *Habenaria* species to the total flowering time of a given *Habenaria* species, and U refers to the proportion of the flowering period that is not shared between two *Habenaria* species.

#### Functional Floral Morphology

To evaluate the interspecific variation of floral traits between the three species, we randomly selected 50–80 plants in 1–2 populations for each species in late July 2017. For each plant, we randomly selected one flower per inflorescence, tagged it, and measured floral structures with a digital caliper (error: 0.01 mm). For floral traits measurement, we followed the method of Tao et al. (2018a) focusing mainly on pollination efficiency-related traits. We measured the following 10 characters: (i) the length and (ii) the width of a lateral petal, (iii) the spur length, (iv) the width of spur opening, (v) the length of the flower (from the highest tip of a lateral petal to the tip of labellum), (vi) the distance between the two pollinia, (vii) the distance between the two stigma lobes, (viii) the distance between the two viscidia, (ix) the length, and (x) the width of a lateral sepal.

### Pollinator Observations

We observed pollinators during night-time in 2016 and 2017 with the help of red-light torches for three species following the observation method of Tao et al. (2018a). Night-time observations were conducted from 19:30 to 22:00 and from 00:00 to 06:30. Three persons walked slowly (around 60 steps per minute) following three pre-set routes (routes were set up in the daytime and one route for each person to do observation at night) within each site by checking each freshly flowering plant for floral visitors with a distance of at least 2 m away from plants. In total, we observed pollinating insects of *H. davidii* (in JQMK, CSZC, and BSZT sites) for 120 h and *H. delavayi* (in YSZ, JLZ, and DBWG sites) for 40 h. Based on the pollinator observations of *H. limprichtii* in Tao et al. (2018a), we did more observations (20 h) in this study but without collecting flower visitors.

Flower visitors were captured with butterfly nets and euthanized in jars with fumes of ethyl acetate (the permission was issued by Lijiang Forest Biodiversity National Observation and Research Station). Freshly killed moths were pinned and photographed (details in Tao et al., 2018a). We measured the length of insect proboscides, width and depth of the thorax, and the width of the moth’s head using digital calipers (error: 0.01 mm). We recorded the number and location of pollinaria attached to the bodies of each moth. The pollinaria of *H. limprichtii* and *H. davidii* are similar in morphology; therefore, we only caught moths after visiting a flower of either species, afterwards we immediately recorded the pollinaria carrying on the body of moth and also checked the visited flowers to see if pollinaria were freshly removed. All moths were sent to the Kunming Institute of Zoology, CAS, for identification, and voucher specimens were deposited in the Kunming Institute of Botany, CAS, Kunming, Yunnan.

#### Pollinator Preference and Ethological Isolation

To test pollinator preferences when a species pair shared the same pollinator, we conducted controlled choice experiments in the field. We randomly dug up bolting inflorescences of *H. limprichtii* (*n* = 15, population YSZ) and *H. davidii* (*n* = 15, population JQMK) and planted them in individual flower pots (diameter = 20 cm, height = 15 cm). When plants were in full bloom, we established a 5 × 6 m^2^ experimental array by placing pots at intervals of 100 cm (corresponding to the minimum distance between plant species found *in situ*). To avoid the influence of prior experience on pollinator choices, we chose an experimental site (Yulong Cun) which is about 5 km away from the nearest locations where *Habenaria* species do not occur naturally. To prevent pollinator visitation prior to the experiment and outside of our pollinator observation periods, we covered each inflorescence with a mesh bag. We recorded moth visits to potted plants for nine nights from 19:30 to 22:00 with the help of red-light torches. During each observation period, we recorded the pollinator identity and foraging route. We also established experimental arrays at Yulong Cun between *H. limprichtii* (*n* = 15, from YSZ) and *H. delavayi* (*n* = 15, from DBWG) following the same general set-up of transplantation and bagging but locating the pots only within 30 cm based on their closest distances *in situ*. We made observations for 10 nights. We did not conduct this experiment for *H. davidii* and *H. delavayi* as they do not share pollinators.

Reproductive isolation due to the likelihood of intra- and inter-specific transitions by foraging pollinators between flowers was calculated following the equation 4A of Sobel and Chen (2014):


(2) RI=1-2×(H/(H+C))

C refers to the proportion of intraspecific transitions and H refers to the proportion of interspecific transitions between flowers in the experimental arrays.

To calculate the constancy of individual pollinators, we used Constancy Index (CI) suggested by Gegear and Thomson (2004):


(3) CI=(c-e)/[c+e-2ce]

where *c* is the actual proportion of intraspecific visits, and *e* is the theoretical proportion of interspecific visits. If *p* is the proportion of visits to one of the plants, then *e = p*^2^ + (1 - *p*)^2^. Possible values range from −1 (random foraging) to 1 (complete constancy).

### Post-pollination Barriers

#### Pollinia–Pistil Interactions

We selected and bagged 35–45 bolting inflorescences of each species (*H. limprichtii, H. davidii*, and *H. delavayi)* to determine whether interspecific pollen germinates and produces pollen tubes that penetrate pistils and enter ovules. As flowers opened, one to three randomly chosen flowers per inflorescence were subjected to either one of the following treatments: (i) self-pollination; (ii) intraspecific cross-pollination, with pollen from a plant growing at least 10 m away from the recipient; and (iii) interspecific pollination. Pistils of hand-pollinated flowers (27–32 pistils for each treatment; see Supplementary Table 5 for detailed information) were harvested 10 days later as fertilization in orchids is often delayed because pollen tubes may remain at the base of styles until megasporogenesis is completed within the ovaries (Arditti, 1992).

We followed the Tao et al. (2018b) protocols for preserving and analyzing pollen tubes in pistils. Pistils were excised, fixed in 3:1 95% ethanol: glacial acetic acid for 12 h, then transferred and preserved in 70% ethanol. In the lab, pistils were softened by sodium sulfite for 2 h. Softened pistils were placed on a glass slide and stained with decolorized aniline blue, and tissues were spread under a coverslip. Each specimen was observed under an epifluorescence microscope (Axio Lab.A1, Zeiss, Oberkochen, Germany). The number of pollen tubes penetrating the style and the number of pollen tubes that entered the ovary were recorded for each pistil.

RI of pollinia–pistil interactions is calculated using Equation 2 (equation 4A of Sobel and Chen, 2014). Here, C refers to the number of pollen tubes that entered ovaries following intraspecific pollination. H refers to the number of pollen tubes entering the ovary following interspecific pollination.

#### Fruit Production

To test for the effect of pollen source (intra- vs. inter-specific) on fruit production, we conducted the same hand-pollination treatments as described earlier but flowers were retained on the inflorescences to allow fruit development (50–88 flowers per treatment; see Supplementary Table 6 for detailed information). Once fruits matured in October and November, we counted and collected all the fruit capsules and stored them in separate envelopes. We judged a flower converted into fruit by squeezing a fruit, if the fruit was dense and turgid, it was recorded as setting fruit, whereas empty fruit remained soft and hollow. However, we also collected these empty fruits to double-check if they contained developed embryos, see below.

Equation 2 was used to calculate the index of RI of fruit production where C refers to fruit production (the number of fruits of each hand pollination treatment) following intraspecific crosses; H refers to fruit set following interspecific crosses.

#### Seed Development

The seeds of each fruit collected from the hand pollination treatments (30–64 fruits per treatment; see Supplementary Table 6 for detailed information) were extracted and placed in a separate Petri dish for assessing seed development. Seed development was examined by scoring approximately 300 seeds in each fruit under an Olympus BX51 microscope (Tokyo, Japan) following the method used in Ren et al. (2014). Seeds were classified as large embryos, small (half-sized) embryos, aborted, and/or empty (no embryos; see Ren et al., 2014 and Tao et al., 2018a).

We used the rate of large embryos to assess seed development, and the Equation 2 was applied, to calculate the RI of the seed development, where C refers to the rate of large embryos derived from intraspecific pollination and H refers to the rate of large embryos resulting from interspecific pollination.

### Total Isolation

Total RI between the three *Habenaria* species was calculated following Sobel and Chen (2014):


(4) $${\text{RI}}_{\text{total}}=1-2\times \frac{\text{S}\times \text{Hs}+\text{U}\times \text{Hu}}{\left(\text{S}\times \text{Hs}+\text{U}\times \text{Uu}\right)+\left(\text{S}\times \text{Cs}+\text{U}\times \text{Cu}\right)}$$

Here, S refers to the proportion of days of the total phenology of each species that overlaps with the flowering period of any of the other two species, and U refers to the proportion of days of the flowering phenology that each species flowers in isolation outside of the flowering period of any of the other two species. H and C represent all components of RI for the interspecific and intraspecific effects for the shared (Hs, Cs) and the unshared (Hu, Cu) period of flowering (see Sobel and Chen, 2014).

To calculate the relative contribution of each reproductive barrier to total isolation (AC_*i*_), the individual strength of the barrier was discounted by the impact of previously acting barriers by subtracting for each index of RI the index of the preceding barrier:


(5) ACi=RI[1,i]-RI[1,i-1]

Finally, we calculated the asymmetry of each barrier as the absolute value of the difference between RI indices for both directions of a given species pair following Lowry et al. (2008).

### Statistical Analysis

We used one-way ANOVA to compare the soil water content among different sites occupied by the three *Habenaria* species. Because significant differences were detected, a least significant difference (LSD) *post hoc* test for multiple comparisons (Hothorn et al., 2008) was used to determine which sites significantly differed in soil moisture.

Morphological variation (including 10 floral traits) between the three *Habenaria* species was visualized in a two-dimensional dispersion diagram with 95% confidence ellipses using principal coordinate analysis (PCoA) in the ‘ape’ packages in R (Paradis and Schliep, 2019). Then, we assessed differences in all floral characters using a one-way ANOVA with Tukey’s HSD *post hoc* tests.

The “G-Test” in the “Desctools” (Signorell, 2016) package was applied to compare the number of cross-visits of moths between *H. limprichtii versus H. davidii*, and *H. limprichtii versus H. delavayi*.

We applied a Kruskal–Wallis analysis of variance for analyzing the effect of cross-type on the proportion of pollen tubes that entered the style, the proportion of pollen tubes penetrating ovaries, and the ratios of large embryos. We then used Dunn’s *post hoc* test (Dinno, 2017) to determine pairwise differences between the former analyses.

Differences in fruit production between the three hand-pollination treatments were analyzed for each *Habenaria* species separately using generalized linear models (GLMs) with binomial error distribution and a logit link function. The model contained pollination treatment as a fixed effect and fruit production as a binary response variable. Then, we evaluate the significance of all GLM models using the ANOVA in the R package “car” (Fox and Weisberg, 2011). We then used a “glht” function in the “multcomp” package (Hothorn et al., 2008) to access pairwise differences for the above analysis.

All data analysis was conducted using the open-source R computational environment (version 4.1.2, R Development Core Team, 2021).

## Results

### Geographic Range and Habitat Differentiation

The three *Habenaria* species are mainly distributed in the Yunnan and Sichuan provinces of southwestern China. We found more than half of the records had at least one species pair co-occurred within a 1 × 1 longitude/latitude grid based on GPS coordinates (52.4%, 44 out of 84 populations) suggesting a substantial overlap in the distributional range (Figure 1). In Lijiang, three species show an intensive co-occurrence, the shortest distances among populations of any of two species ranged from 1 m to 1.9 km (Supplementary Figure 1).

In the Lijiang populations, the soil water content varied significantly among habitats of the different species (one-way ANOVA, *F* = 271, d.f. = 2, *p* < 0.001) but was homogeneous among the habitats within species (all *p* > 0.05). The soil water content of *H. limprichtii* sites was significantly greater than that of the sites where *H. davidii* (*P* < 0.001) and *H. delavayi* (*P* < 0.001) were found. The soil water content of *H. delavayi* habitats was greater than that of *H. davidii* (*P* < 0.001) (Figure 3).

### Phenological Isolation

Flowering phenology overlap of the three *Habenaria* species varied among different species pairs. *Habenaria limprichtii* and *H. davidii* showed a substantial flowering overlap (*S* = 0.7377) for most of their flowering periods. In contrast, *H. delavayi* flowered two weeks earlier than the former two species (*S* = 0.3833 when *H. delavayi* compared with *H. limprichtii*, *S* = 0.3026 when *H. delavayi* compared with *H. davidii*; Supplementary Table 2).

Reproductive isolation resulting from differences in flowering phenology was weak between *H. limprichtii* and *H. davidii* (RI_lim♀dav♂_ = 0.026, RI_dav♀lim♂_ = 0.011) but strong among the other two species pairs, *H. limprichtii* and *H. delavayi* (RI_lim♀del♂_ = 0.605, RI_del♀lim♂_ = 0.559) and *H. davidii* and *H. delavayi* (RI_dav♀del♂_ = 0.667, RI_del♀dav♂_ = 0.558).

### Phenotypic Divergence

**FIGURE 4:**
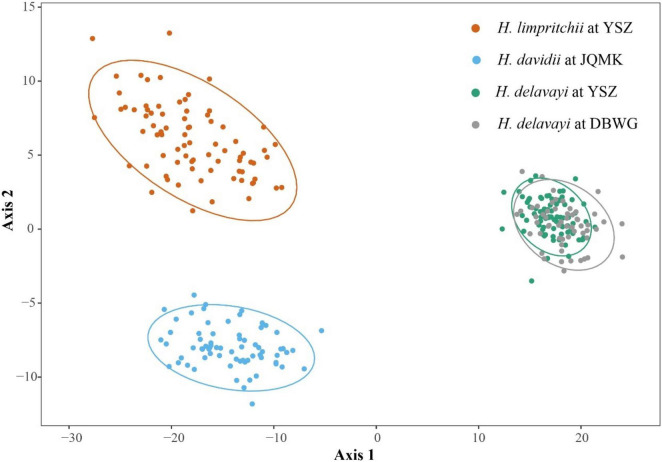
Principal coordinate analysis (PCoA) based on the first two principal coordinate axes of ten floral traits of three *Habenaria* species with ellipse type assumed using a multivariate t-distribution with a 95% confidence level.

Floral characteristics of the three *Habenaria* species are summarized in Supplementary Table 3. The PCoA diagram showed no overlap among the three populations representing each one of the species, and they occupied different morphometric spaces (Figure 4). The PCoA including 10 flowering traits of the three *Habenaria* species showed that the first principal coordinate axes (represented by the flower length, lateral sepal length, and lateral petal length) and the second principal coordinate axes (represented by the spur length and the distance between the two pollinia) explain 98% of the total variation of floral traits (Supplementary Table 4). *Habenaria limprichtii* had a wider spur entrance than the other two species among which there was no significant difference. The length of the lateral sepal was similar between *H. limprichtii* and *H. davidii* but significantly reduced in *H. delavayi* to about one quarter in length. All other morphological traits varied significantly among all the three species (Supplementary Table 3). However, the phenotypic differences between *H. limprichtii* and *H. davidii* were less marked than those of *H. delavayi.*

### Pollinator Observations

We further confirmed the observation by Tao et al. (2018a) that *Deilephila elpenor* subsp. *lewisii* and *T. intermixta* were the dominant pollinaria vectors of *H. limprichtii* (Figures 2C,D). During our observations of *H. davidii*, we caught 35 insects belonging to 5 different species (in decreasing abundance: *D. elpenor* subsp. *lewisii*, *Agrius convolvuli*, *Theretra nessus*, *Eupanacra mydon*, and an *Apamea* sp.). Only *D. elpenor* subsp. *lewisii* (proboscis length: 28.19 ± 1.71 mm, *n* = 10) carried several pollinaria on its compound eyes (Figure 2G). We observed *Agrius convolvuli* with long proboscis visiting flowers of *H. davidii* but without carrying any pollinaria. *Habenaria davidii* shared one hawkmoth species, *D. elpenor* subsp. *lewisii*, with *H. limprichtii*. The pollinaria of *H. limprichtii* attached to the entire area of the eyes, but the pollinaria of *H. davidii* were attached to the posterior lower margin of the eyes.

We collected 30 moths belonging to 6 species in the family Noctuidae (in decreasing abundance: *Trichoplusia intermixa, D. elpenor* subsp. *lewisii*, *Odontopera* sp., *Apamea* sp., *Plusidia imperatrix*, and *Panchrysia ornata*) foraging on flowers of *H. delavayi*. Pollinaria were attached to the bases of the moth proboscides of *T. intermixta* (proboscis length: 15.26 ± 1.07 mm, *n* = 14; Figure 2K) and one specimen of the *Apamea* sp., (Figure 2L).

*Habenaria delavayi* and *H. limprichtii* shared one pollinator species, *T. intermixa*, but the pollinaria of *H. limprichtii* were carried on the lateral-ventral side of the thorax and the femora of middle and hind legs (Figure 2D). No shared pollinators were observed between *H. davidii* and *H. delavayi*.

### Pollinator Preferences and Ethological Isolation

For the species pair *H. limprichtii* and *H. davidii*, we recorded 11 foraging bouts by moths making a total of 34 flower visits within the experimental arrays. The number of intraspecific cross-visits (*n* = 30; 16 lim → lim, 14 dav → dav) was significantly higher than the number of interspecific cross-visits (*n* = 4, 3 lim → dav, 1 dav → lim; Table 1; *G* = 22.50, d.f. = 1, *p* < 0.001; Table 1). Only *D. elpenor* subsp. *lewisii* was observed to make cross-visits between these two orchid species. We found that the strength of reproductive isolation due to foraging patterns by the primary pollinator of *H. limprichtii*, RI = 0.882 and *H. davidii*, is RI = 0.647.

**TABLE 1:** Interspecific *versus*. intraspecific foraging bouts by hawkmoths.

Pollinators	Interspecific transitions/total transitions	No. of floral transitions				CI
**a) *H. limprichtii* versus *H. davidii***						
		*lim*→*lim*	*dav*→*dav*	*lim*→*dav*	*dav*→*lim*	
*Deilephila elpenor*	2/7	2	14	3	1	0.70 ± 0.69
*Trichoplusia intermixta*	0/4	14	0	0	0	1.00 ± 0.00
Total	2/11	16	14	3	1	
**b) *H. limprichtii* versus *H. delavayi***						
		*lim*→*lim*	*del*→*del*	*lim*→*del*	*del*→*lim*	
*Deilephila elpenor*	0/3	8	0	0	0	1.00 ± 0.00
*Trichoplusia intermixta*	2/6	2	13	2	2	0.86 ± 0.20
Total	2/9	10	13	2	2	

*The constancy index (CI, mean ± SD) is shown for H. limprichtii versus H. davidii (lim vs. dav) and H. limprichtii versus H. delavayi (lim versus del).*

We recorded 9 foraging bouts by moths resulting in 27 flower visits in the experimental array of *H. limprichtii* and *H. delavayi*, including 23 intraspecific transitions (10 lim → lim, 13 del → del) and 4 interspecific transitions (2 lim → del, 2 del → lim; Table 1). The number of intraspecific cross-visits was significantly higher than that of interspecific visits (*G* = 14.78, d.f. = 1, *P* < 0.001). *Thereta intermixta* was the only pollinator that alternately visited both species. The strength of reproductive isolation for *H. limprichtii* and *H. delavayi* was RI = 0.667 and RI = 0.733, respectively.

### Pollinia–Pistil Interactions

**FIGURE 5:**
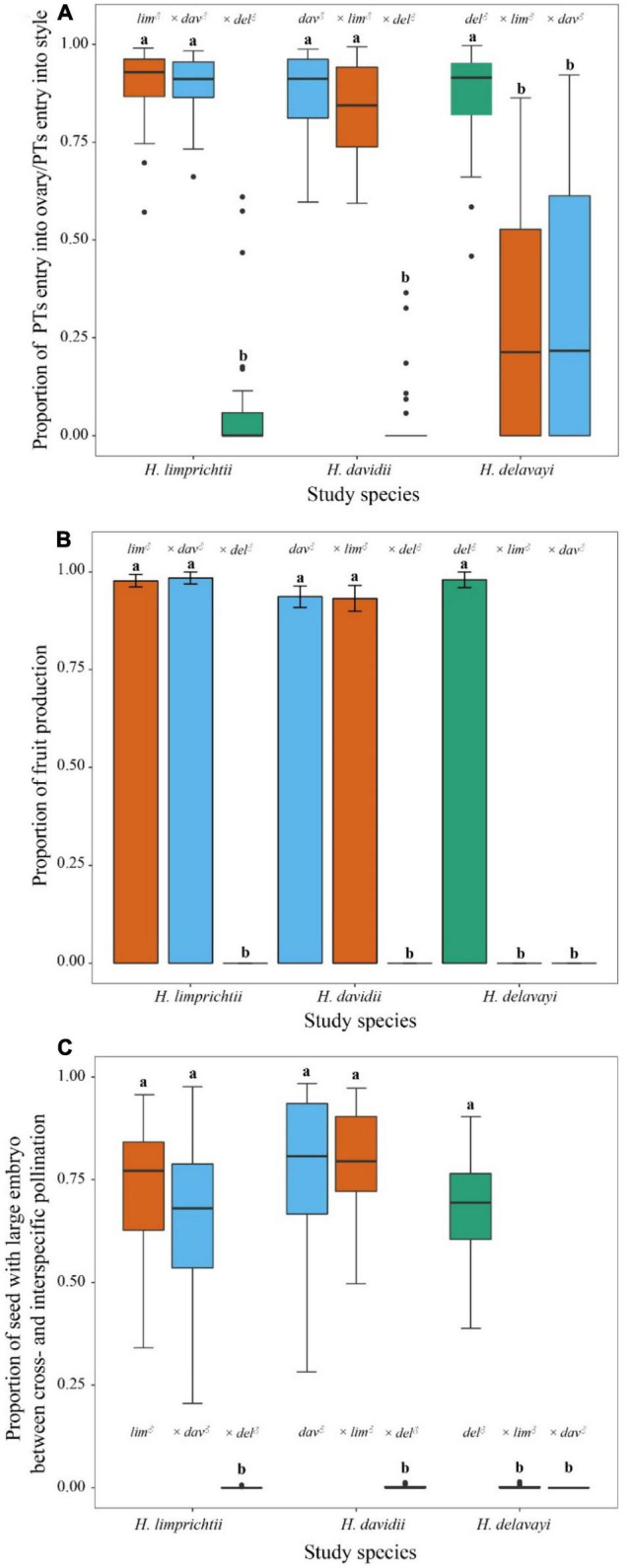
The effect of intra- and interspecific cross-type on **(A)** the proportion of pollen tubes entering into styles and ovary; **(B)** a number of fruits produced; and **(C)** the proportion of seed with large embryos among three *Habenaria* orchids. Lower case letters indicate significant differences at *p* < 0.001. *lim*, *Habenaria limprichtii*, *dav*, *H. davidii*, *del*, *H. delavayi*.

The interspecific crosses between *H. limprichtii* and *H. davidii* showed the same proportion of pollen tubes entering the ovary as compared with the intraspecific crosses for the two species, and there was no difference whether they served as donors or as recipients (*p* > 0.05). However, the rate at which pollen tubes entered an ovary was significantly lower when *H. delavayi* served as either female or male parent (all *p* < 0.001; Figure 5A, Supplementary Figure 2 and Supplementary Table 5).

With respect to pollen tube-ovary compatibility, the RI indices were low for the species pair *H. limprichtii* and *H. davidii* as female parents with RI_lim♀dav♂_ = −0.006 and RI_dav♀lim♂_ = 0.029, respectively. In contrast, the RI values including *H. delavayi* for either male or female function were strong: RI indices for the species pair *H. limprichtii* and *H. delavayi* as female parents were RI_lim♀del♂_ = 0.798 and RI_del♀lim♂_ = 0.500. For the species pair *H. davidii* and *H. delavayi*, the RI indices were RI_dav♀del♂_ = 0.913 and RI_del♀dav♂_ = 0.450.

### Fruit Production

All intraspecific crosses of the three species and interspecific crosses between *H. limprichtii* and *H. davidii* produced a fruit set ranging between 93 and 98% while when *H. delavayi* served as the female or male parent for interspecific crosses, all treated flowers failed to produce dense and turgid fruits (Figure 5B).

For fruit production, the RI was low for *H. limprichtii*, RI = −0.004 and *H. davidii* RI = 0.002 for female parents. Conversely, the RI of this barrier for the *H. limprichtii* and *H. delavayi* pairing, and the *H. davidii* and *H. delavayi* pairing, as female parents were RI = 1.

### Seed Development

Similar to fruit production, when *H. delavayi* served as the female or male parent for interspecific crosses, all the treated fruits contained very few embryos, which were significantly less than any other hand-pollination treatments (all *p* < 0.001, Figure 5C, Supplementary Figure 3, and Supplementary Table 6). Reproductive isolation as female parents *via* seed development for *H. limprichtii was* RI = 0.044 and *H. davidii*, RI = −0.013. For *H. limprichtii* and *H. davidii* as female parents when pollinated with pollen from *H. delavayi*, the RI value equaled 1.

### Total Reproductive Isolation and Relative Contributions of Each Isolating Barrier

The total isolation strength of species pair lim♀del♂, del♀lim♂, dav♀del♂ and del♀dav♂ summed to complete reproductive isolation with RI_*total*_ = 1. The total isolation strength of species pair lim♀dav♂ was 0.895. The weakest isolation strength was found for the species pair dav♀lim♂ with RI_*total*_ = 0.660.

The strength of each isolation barrier ranged from −0.013 to 1. The highest values of reproductive isolation corresponded to pollinator visitation and pollinia-pistil interactions (pollen tube development and ovary penetration) when compared with the other isolating barriers. The lowest values of reproductive isolation corresponded to fruit production. Evidently, the pre-pollination barriers are prominent and strong.

**FIGURE 6:**
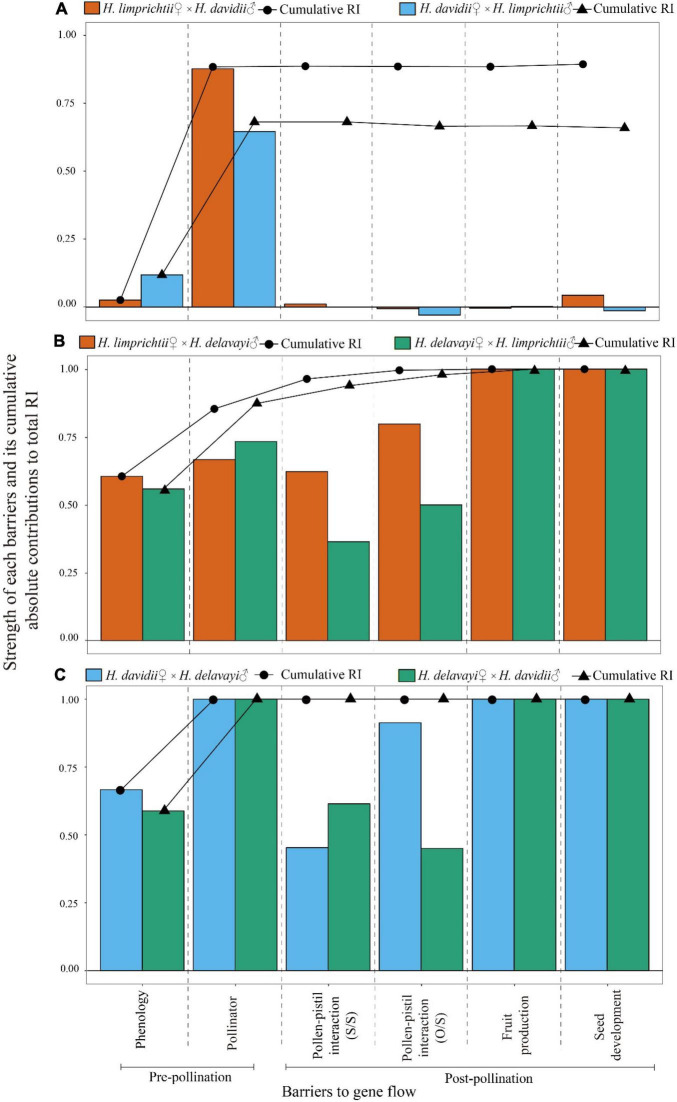
Absolute contributions of six sympatric barriers to total isolation in reciprocal crosses between each of the three *Habenaria* species pairs. **(A)** *Habenaria limprichtii* and *H. davidii*, **(B)** *H. limprichtii* and *H. delavayi*, **(C)** *H. davidii* and *H. delavayi* following the method described in Sobel and Chen (2014). The line graph represents the cumulative contribution to RI of a mechanism after accounting for each of the investigated previous mechanisms. S/S, number of pollen tube entry into style/pollen germinated on the stigma; O/S, number of pollen tube entry into ovary/style.

The relative contributions of the different sequential barriers to total RI varied from 0.682 to 1 for pre-pollination barriers and from −0.022 to 0.146 for post-pollination barriers. The contribution of pre-pollination isolation to total isolation was significantly higher than that of post-pollination isolation barriers (χ^2^ = 8.36, d.f. = 1, *P* < 0.01; see Figure 6).

## Discussion

### Ecogeographic Isolation

To restrict the production of viable hybrid offspring, most plant species employ an array of different isolation mechanisms. Each isolation factor can only suppress what was not blocked by the former isolation stage during the reproductive cycle (Lowry et al., 2008; Widmer et al., 2009; Baack et al., 2015). Since geographic isolation occurs first in the life history of plants, it is generally considered to be the first barrier to operate in limiting interspecific hybridization (Lowry et al., 2008; Sobel et al., 2010; Glennon et al., 2012; Sobel and Streisfeld, 2015; Arida et al., 2021). Although we have not conclusively quantified geographic isolation here, our herbaria investigations and field observations suggest that at a large geographic scale, each of these three species does not clearly occupy a distinct geographic region. In the biodiversity hotspot of the Hengduan Mountains, closely related species from the same genus commonly co-exist and do not show clear differentiation in distributional ranges at a large geographical scale (Liang et al., 2018).

As a consequence of weak ecogeographic isolation at a larger geographic scale, microhabitats can be expected to be of major importance in segregation among our three species in the focal area in Lijiang, and we did find soil water content varied among species. Although the distance between these microhabitats (1 m–1.9 km; Supplementary Figure 1) was within the foraging distance of a hawkmoth (possibly within 2.5 km; Skogen et al., 2019), microhabitat difference may not be able to act as a barrier for pollen dispersal of our species pairs sharing pollinator species. In a previous study by Tao et al. (2018b) in the same region, they also find the white and pink floral morph in the orchid *Spiranthes sinensis* lived in different microhabitats with contrasting soil water content. *Habenaria* species, similar to other terrestrial orchids in the Orchidoideae, typically grow in habitats that are moist to dry during the flowering season (Tao et al., 2018b; Sakaguchi et al., 2019). Future research should investigate other factors that may contribute to habitat variation, such as variation in light intensity, maximum temperature, inorganic nitrogen, and heavy metal elements. In addition, reciprocal transplant experiments in combination with experimental manipulation of environmental factors could test the possibility of microhabitat adaptation for divergence and maintenance of these sympatric species assemblage.

### Phenological Isolation

Flowering phenology is a crucial pre-pollination isolation mechanism in which interspecific pollen transfer among sympatric species is prevented by species-specific flowering times (Grant, 1994; Martin and Willis, 2007). In this study, the blooming periods of *H. limprichtii* and *H*. *davidii* have widely overlapped (phenological isolation was very low) suggesting a limited barrier to interspecific cross-pollination. In contrast to our study, Zhang and Gao (2017) suggest that in the southeastern region of Yunnan, flowering phenology between both species acts as the primary isolating factor. The study populations of Zhang and Gao (2017) were located in subtropical forests at a lower latitude and lower elevation compared with our study site which is a sub-alpine meadow at high elevation. This difference in divergence of flowering time between two species in both geographic locations might be due to the difference in latitude and elevation. Importantly, these contrasting results suggest the context-dependence and spatial variation in the contribution of the same isolating barrier and highlight the importance of geographic replica of reproductive isolation studies. Geographic variation in reproductive isolation among different species has been previously documented (Barnard-Kubow and Galloway, 2017; Kay et al., 2019). For example, the flowering time of Iberian *Arabidopsis thaliana* showed substantial variation within- and among- population, suggesting that flowering time is likely to be under divergent selection (Méndez-Vigo et al., 2013). But studies quantifying multiple reproductive isolation barriers among closely related species covering the full geographic ranges of these species are lacking and, therefore, warrant further study.

Pollinator interactions may drive divergence in flowering time (Rafferty and Ives, 2012; Chapurlat et al., 2020). For example, *H. delavayi* began to flower about two weeks earlier than *H. limprichtii* and *H*. *davidii* and reduced the competition for pollinators at least when co-occurring with *H. limprichtii* which it shares a pollinator. Phenotypic selection studies in combination with manipulation of the pollination environment are needed to clarify whether pollinators act as selective agents contributing to divergence in flowering time (Chapurlat et al., 2020).

### Pollinator Isolation

Floral isolation may be mediated by the attraction of different suites of pollinators or by the different placement of pollinia when pollinators are shared (Grant, 1994; Maad and Nilsson, 2004; Sun et al., 2011; Zhang and Gao, 2017). In our study, differentiation in the pollinator assemblage among the species pair *H*. *davidii* and *H. delavayi* can possibly effectively ensure pre-pollination reproductive isolation, although we need to keep in mind that, in this study, we did not perform choice experiments for this species pair since we never found any hawkmoths visited *H. delavayi*. Adaptive radiation to different pollinators among sympatric species in the same lineage is displayed among terrestrial orchid genera such as *Cypripedium* in temperate Asia (Edens-Meier and Bernhardt, 2014; Edens-Meier et al., 2014), *Disa* and *Satyrium* in southern Africa (Johnson, 1997, 2014), and *Platanthera* in the Azorean Islands (Bateman et al., 2013).

Our pollinator choice experiment with *H. limprichtii* and *H*. *davidii* showed that intraspecific floral transitions of insects were significantly higher than interspecific floral transitions. This indicates that ethological isolation due to pollinator foraging behavior is very strong between this species pair even when species share the same pollinator species. Tao et al. (2018a) suggested that the distance between two viscidia is subject to selection to attach pollinaria on the eyes of hawkmoth in *H. limprichtii*. Although the distance between the two viscidia of *H. davidii* was much shorter than in *H. limprichtii*, we found that the pollinaria of both *Habenaria* species are carried on the eyes of the *D. elpenor* subsp. *lewisii*. Although we also noticed that the areas of eyes for pollinaria attachment for both species were different, whether such a small difference could cause strong mechanical isolation in this species pair requires further investigation. Additionally, it is not easy to distinguish the pollinaria of both species when they are carried in the eyes of hawkmoth. To avoid misidentification of pollinaria for both species to the lowest level, we only collected the hawkmoth visiting the flowers of either species. Our results tend to support that reproductive isolation between *H. limprichtii* and *H*. *davidii* is primarily based on pollinator foraging choice. Evidently, a more extensive investigation with multiple natural populations with potential different pollinator assemblages is required in order to fully understand which functional traits do effectively drive pollinator foraging preferences promoting ethological and mechanical RI. We should keep in mind that floral scent is the main attractive signal for the nocturnal moth pollination system (Raguso et al., 1996; Tao et al., 2018a). Previously, Tao et al. (2018a) reported that the floral scent of *H. limprichtii* was dominated by two aromatic benzenoid compounds, benzyl acetate, and benzaldehyde. Based on human noses, we do notice that the floral scent of flowers of *H. davidii* is different from *H. limprichtii* which may be the main reason causing the foraging preferences of hawkmoth. This requires further investigation.

Although *H. limprichtii* shared a second pollinator with *H. delavayi*, the noctuid moth *T. intermixta*, pollinaria of the two orchid species were attached to different body parts as a result of matching the length of the floral spur and the length of the proboscis of moth reinforcing reproductive isolation by mechanical pollination (Sensu Grant, 1994). This mode of isolation may be more common in terrestrial orchids, particularly in Orchidioideae, than previously documented studies in *Platanthera* (Nilsson, 1983; Maad and Nilsson, 2004) and other orchid genera (reviewed in Schiestl and Schlüter, 2009). The matching between the length of spurs of orchids and the length of proboscides of pollinating moths is the key to such a mechanical isolation barrier.

### Post-pollination Isolation

Pollen-pistil interactions (interspecific incompatibility) also play an important role in blocking interspecific fertilization between plant species (Dellòlivo et al., 2011). In our study, we only found a significantly lower number of interspecific pollen tubes penetrating the stigmatic surface and the ovary when *H. delavayi* served as either female or male parent. However, because few interspecific tubes have always been able to enter the ovaries, interspecific isolation at this stage remained incomplete. Furthermore, we cannot rule out population effects because we did not replicate more populations in the crosses. In the two floral color morphs of *Spiranthes sinensis* complex, Tao et al. (2018b) observed that prevention of intra-morph fruit formation occurred at a later stage after pollen tubes entered the ovary. In our *Habenaria* species, we also found stronger effects on fruit set and embryo development than at the earlier stage of pollen tube growth similar to *S. sinensis*. Additionally, Pellegrino et al. (2005) also found that post-pollination barriers were responsible for differential fertility among color morphs of Southern Italy populations of the deceptive orchid *Dactylorhiza sambucina*. The post-pollination barriers warrant more investigations in the orchids.

In orchids, fruit and seed development play a very important role in maintaining species boundaries when species pairs show weak pre-pollination isolation barriers (Cozzolino et al., 2005; Cozzolino and Scopece, 2008). In our study, reciprocal crosses between *H. limprichtii* and *H*. *davidii* produced a high fruit set and a high percentage of large embryos, suggesting that the post-pollination isolation mechanism at the pollen-pistil interaction stages between these two species was weak or absent. However, because we did not check late postzygotic barriers, i.e., seed germination and seedling growth of hybrid seeds; therefore, we still do not know if the other postzygotic barriers play roles in their total isolation. In contrast, results of our cross- and intraspecific-pollination treatments showed that post-pollination barriers were far more important in the two species pairs *H. limprichtii* and *H. delavayi*, and, *H*. *davidii* and *H. delavayi* based on significant declines in fruit set and embryonic development.

### Contribution of Pre- and Post-pollination Isolation to Total Isolation

In most orchids, pre-pollination isolating mechanisms have been found to be strong while post-pollination barriers often contribute less to total isolation (Whitehead and Peakall, 2014; Baack et al., 2015; Sun et al., 2015). Pre-pollination barriers based on pollination systems canalizing pollinator diversity and mating systems may evolve more rapidly and ultimately become far stronger and more effective than later evolving post-pollination barriers (Costa et al., 2007; Rieseberg and Willis, 2007; Lowry et al., 2008). This is congruent with the species pair *H. limprichtii* and *H. davidii* which are sisters in the same clade. Interspecific isolation between this species pair appears to be based primarily on pre-pollination barriers, especially pollinator preference, but the total isolation is not complete (RI_*total*_ = 0.660). Because we did not find any potential hybrids for this species pair in our populations and our floral trait analysis also showed distinct morph space for each species, we suspect that intrinsic and extrinsic late postzygotic mechanisms such as hybrid sterility may play roles for maintain their species boundary. Two species grow in distinct wet and dry micro-habitats, such habitat preference may cause hybrid seeds germination failure, and this warrants further investigations.

The strength of pre-pollination isolation was strong but not sufficient between the sympatric pairs, *H. limprichtii* and *H. delavayi*, as well as *H. davidii* and *H. delavayi* that instead showed more effective post-pollination barriers between them. That is, their reproductive isolation depends additionally on post-pollination barriers. Thus, both pre- and post-pollination barriers are required to effectively prevent hybridization and maintain the integrity of the two co-existing species. Liang et al. (2018) argued that floral isolation based on pollen placement on pollinators in the genus *Pedicularis* was crucial to avoid interspecific pollen transfer, and post-pollination barriers may play even larger roles for currently established populations of co-blooming and sympatric species.

In all, based on our limited species pairs, our study supports the hypothesis of the positive association between phylogenetic relationship and reproductive isolation magnitude. Indeed, more studies pondering both pre- and post-pollination isolation barriers are required to fully assess the generality of this relationship by more species pairs with different distant phylogenetic relationships for the species-rich genus, *Habenaria*.

## Conclusion

In this study, we quantified multiple pre- and post-pollination reproductive barriers among three congeneric sympatric orchids and calculated their relative contribution to total reproductive isolation. Our results showed that pollinator isolation is the most important isolating barrier to maintaining the boundaries among the three studied *Habenaria* species. Moreover, our study confirms that reproductive isolation between *H. limprichtii* and *H. delavayi* is a result of a combination of pre-and post-pollination barriers. Habitat differentiation based on soil water content appeared to be of major importance for speciation or maintaining species integrity, but cannot stop pollinator-mediated pollen dispersal in our study system. This study supports the positive association between phylogenetic relationship and reproductive isolation magnitude and highlights the importance of a combination of pre-and post-pollination barriers for species co-existence in Orchidaceae.

## Data Availability Statement

The original contributions presented in this study are included in the article/Supplementary Material, further inquiries can be directed to the corresponding author/s.

## Author Contributions

Z-XR and HW designed the study. H-PZ, Z-BT, and Z-XR conducted the fieldwork. H-PZ, Z-BT, JT, MS, and DS analyzed and discussed the data. H-PZ drafted the first version. All authors revised and discussed subsequent versions.

## Conflict of Interest

The authors declare that the research was conducted in the absence of any commercial or financial relationships that could be construed as a potential conflict of interest.

## Publisher’s Note

All claims expressed in this article are solely those of the authors and do not necessarily represent those of their affiliated organizations, or those of the publisher, the editors and the reviewers. Any product that may be evaluated in this article, or claim that may be made by its manufacturer, is not guaranteed or endorsed by the publisher.
