# Supplementary material for: Reproductive Isolation Among Three Nocturnal Moth-Pollinated Sympatric Habenaria Species (Orchidaceae)
Source: Front Plant Sci. 2022 Jun 22;13:908852. doi: 10.3389/fpls.2022.908852 (PMC9257206; doi:10.3389/fpls.2022.908852)
Supplement: Supplementary file 1 [file Data_Sheet_1.docx]

# Reproductive isolation among three nocturnal moth-pollinated sympatric *Habenaria* species (Orchidaceae)

Hai-Ping Zhang^1, 2†^, Zhi-Bin Tao^1, 3†^, Judith Trunschke^1^, Mani Shrestha^4^, Daniela Scaccabarozzi^5, 6^, Hong Wang^1, 2, 7*^, Zong-Xin Ren^1, 2, 7*^

*^1^CAS Key Laboratory for Plant Diversity and Biogeography of East Asia, Kunming Institute of Botany, Chinese Academy of Sciences, Kunming 650201, China*

*^2^University of Chinese Academy of Sciences, Beijing, China*

*^3^ CAS Key Laboratory of Aquatic Botany and Watershed Ecology, Wuhan Botanical Garden, Chinese Academy of Sciences, Wuhan 430074, China*

*^4^Department of Disturbance Ecology, Bayreuth Center of Ecology and Environmental Research (BayCEER), University of Bayreuth, Bayreuth, 95447, Germany*

*^5^School of Pharmaceutical Science and Technology, Tianjin University, Tianjin 300072, China*

*^6^School of Molecular and Life Sciences, Curtin University, Bentley 6102, Western Australia*

*^7^Lijiang Forest Biodiversity National Observation and Research Station, Lijiang 674100, China*

Running title: Reproductive isolation in sympatric *Habenaria* orchids

Corresponding authors: Zong-Xin Ren ([renzongxin@mail.kib.ac.cn](mailto:renzongxin@mail.kib.ac.cn)) and Hong Wang ([wanghong@mail.kib.ac.cn](mailto:wanghong@mail.kib.ac.cn))

^†^ These authors have contributed equally to this work

# Supplemental files

**
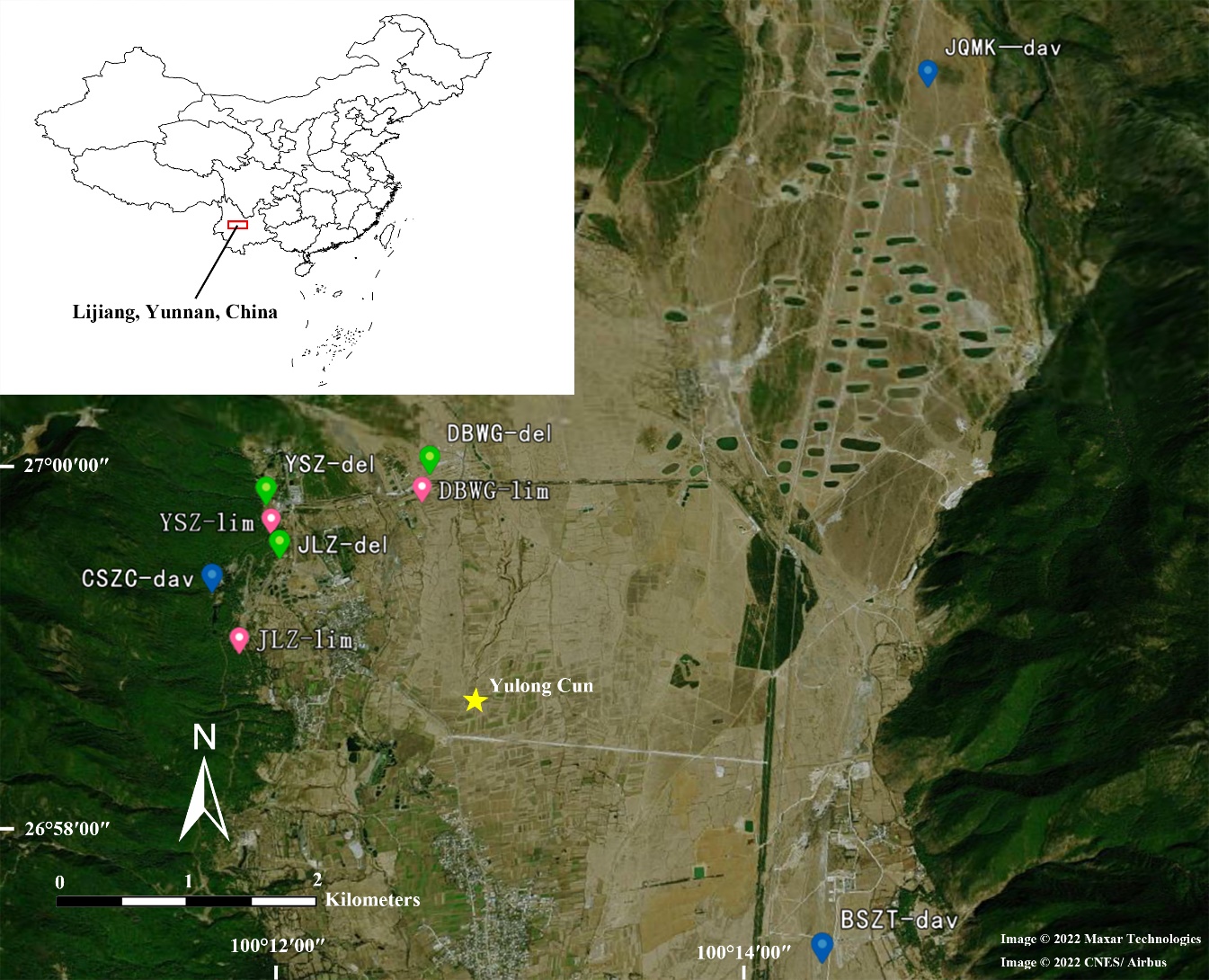
**

**Figure S1.** The distribution ranges of three *Habenaria* species, *Habenaria* *limprichtii*, *H. davidii*, *H. delavayi*, in the study sites. Image from Google Earth (<http://earth.google.com/>). YSZ, JLZ, DBWG, JQMK, BSZT and CSZC are the populations of three *Habenaria* species. The shortest distances for populations of any of two species was from 1 m (at YSZ for *H. limprichtii* and *H. delavayi*) to 1.9 km (from JLZ-lim to DBWG-del). The highest distance among populations within species is 8.2 km. *lim* = *H. limprichtii*, *dav* = *H. davidii*, *del* = *H. delavayi*.


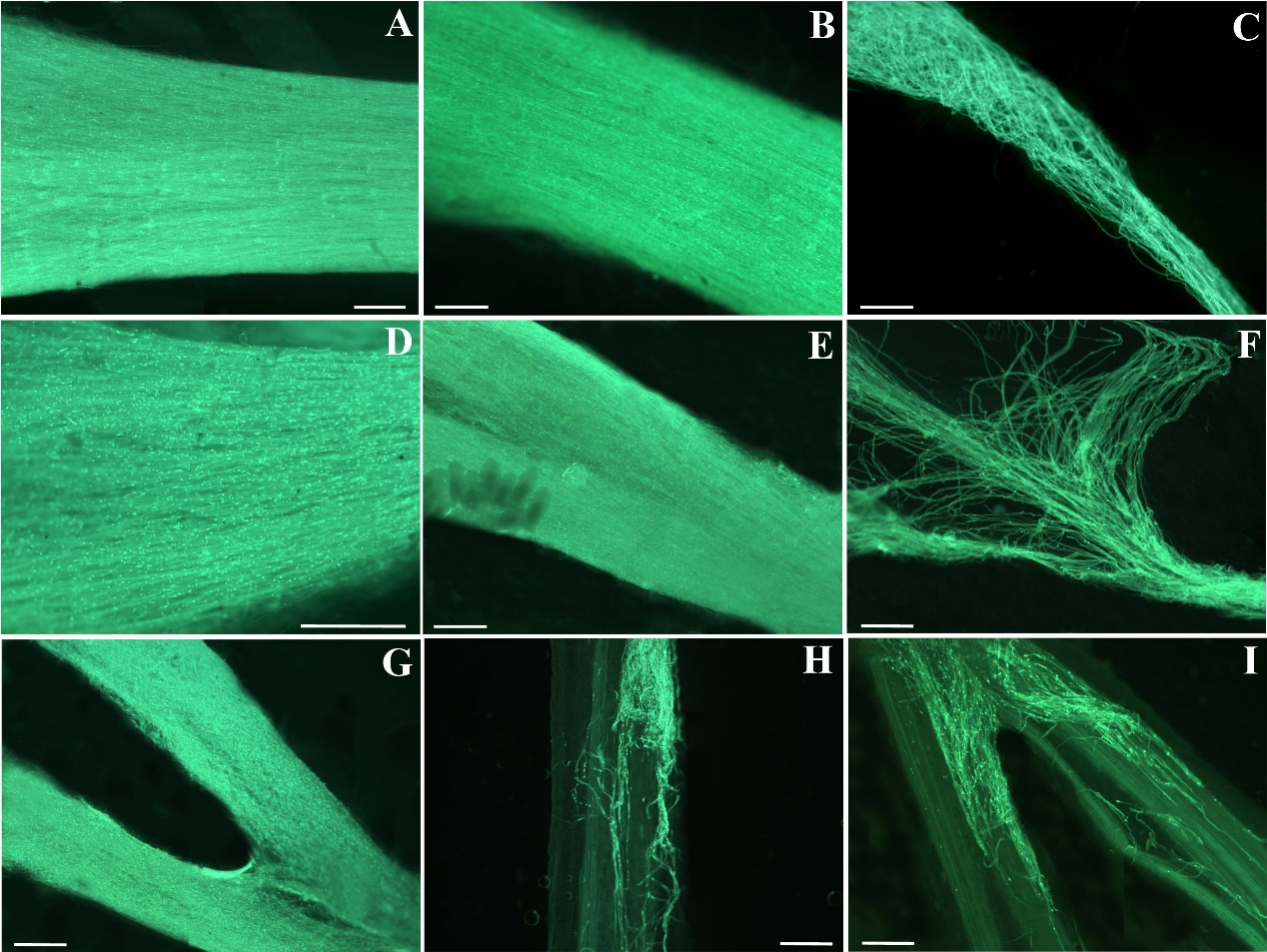


**Figure S2.** Pollen tubes growing in the styles of three *Habenaria* species 10 days after intra- and interspecific cross-pollinations. **(A-C)** *H. limprichtii* as recipient. **(A)** intraspecific cross-pollination. **(B)** *H. davidii* as donor. **(C)** *H. delavayi* as donor. **(D-F)** *H. davidii* as recipient. **(D)** intraspecific cross-pollination. **(E)** *H. limprichtii* as donor. **(F)** *H. delavayi* as donor. **(G-I)** *H. delavayi* as recipient. **(G)** intraspecific cross-pollination. **(H)** *H. limprichtii* as donor. **(I)** *H. davidii* as donor. (A, B, D, E, G) pollen tubes reach the style. (C, F, H, I) pollen tube is arrested in the lower portion of the stigma. Scale bar = 200 μm. All pictures are microscope pictures under an epifluorescence microscope (Axio Lab.A1, Zeiss, Oberkochen, Germany). Except that the magnification of D is 20 times, and the other images are magnified by 10 times.


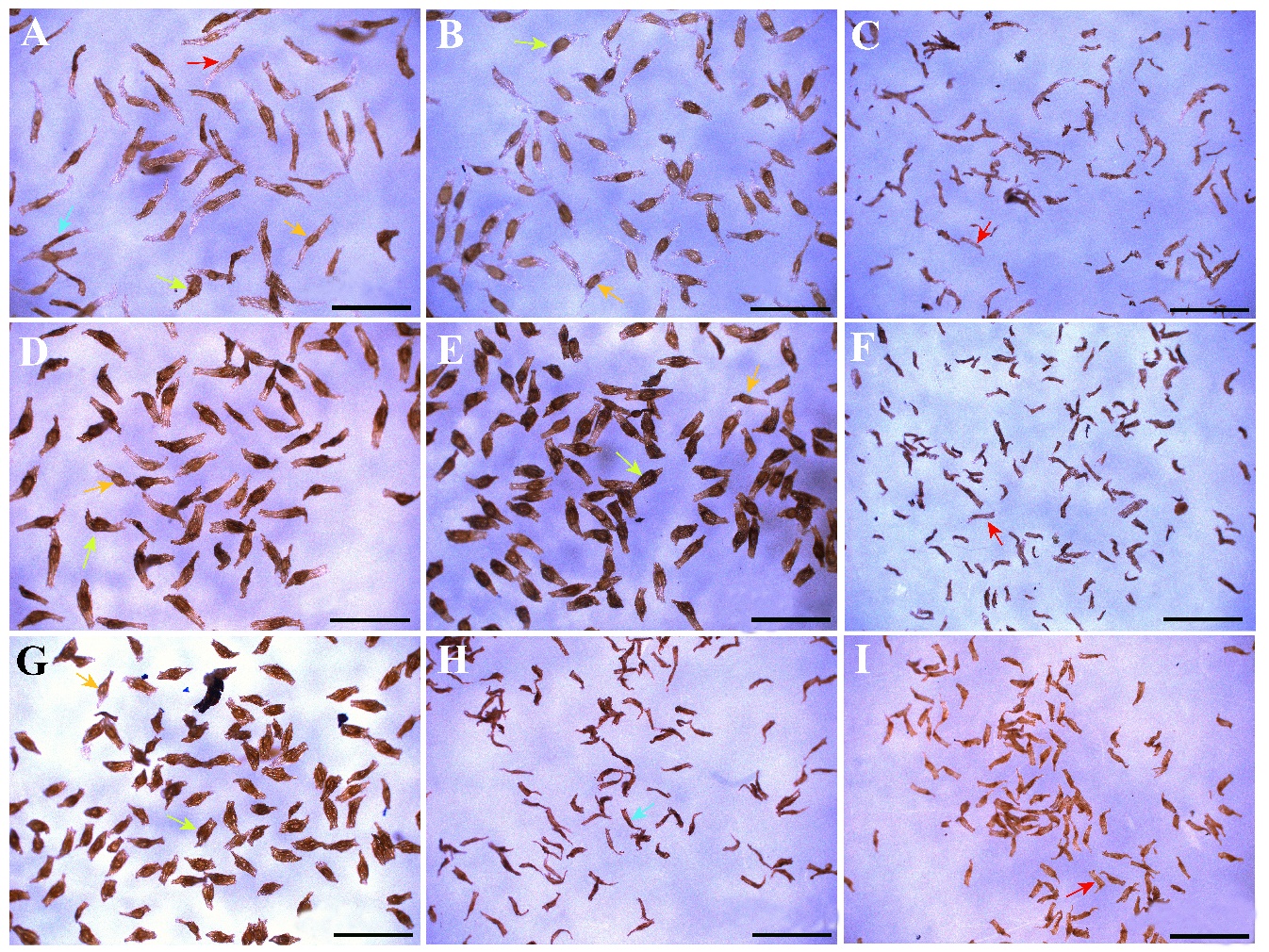


**Figure S3.** Seed development of the intra- and inter-specific cross-pollinated fruits of the three *Habenaria* species. **(A-C)** *H. limprichtii* as recipient. **(A)** intraspecific cross-pollination. **(B)** *H. davidii* as donor. **(C)** *H. delavayi* as donor. **(D-F)** *H. davidii* as recipient. **(D)** intraspecific cross-pollination. **(E)** *H. limprichtii* as donor. **(F)** *H. delavayi* as donor. **(G-I)** *H. delavayi* as recipient. **(G)** intraspecific cross-pollination. **(H)** *H. limprichtii* as donor. **(I)** *H. davidii* as donor. Scale bar = 500 μm. Seeds were classified into four categories: large embryo (indicated by the yellow arrows), small embryo (half sized, indicated by the orange arrows), aborted embryo (indicated by the cyan arrows), and/or empty (no embryos, indicated by the red arrows).

**Table S1.** The Geographic Coordinate data of three *Habenaria* species, from different herbarium, GBIF, and our own exploration data.

| **Species** | **Source** | **GBIF ID** | **Latitude (decimal degree)** | **Longitude (decimal degree)** | **Institution code** |
| --- | --- | --- | --- | --- | --- |
| *H. limprichtii* | GBIF | 919506233 | 21.475834 | 101.56752 | PE |
| *H. limprichtii* | Our own data (LWS) |  | 24.75051667 | 102.8934528 | KUN |
| *H. limprichtii* | GBIF | 1304083203 | 25.012526 | 102.700013 | KUN |
| *H. limprichtii* | GBIF | 919554213 | 25.020615 | 98.490007 | PE |
| *H. limprichtii* | GBIF | 919554259 | 25.275367 | 103.003972 | PE |
| *H. limprichtii* | Our own data (KM) |  | 25.36815833 | 102.8117278 | KUN |
| *H. limprichtii* | GBIF | 1304083183 | 25.584234 | 100.222124 | KUN |
| *H. limprichtii* | GBIF | 147033305 | 25.6333 | 100.167 | A |
| *H. limprichtii* | GBIF | 615329878 | 25.67 | 100.13 | GMBA |
| *H. limprichtii* | GBIF | 2417755942 | 25.7 | 104.46 | PE |
| *H. limprichtii* | GBIF | 919473783 | 26.453601 | 99.416689 | PE |
| *H. limprichtii* | GBIF | 1304083198 | 26.56023 | 100.17643 | KUN |
| *H. limprichtii* | Our own data (JC) |  | 26.60876389 | 99.84855278 | KUN |
| *H. limprichtii* | GBIF | 1304083154 | 26.876096 | 100.229945 | KUN |
| *H. limprichtii* | This study (YSZ) |  | 26.99911111 | 100.1994472 | KUN |
| *H. limprichtii* | GBIF | 1998762049 | 27 | 100 | GH |
| *H. limprichtii* | GBIF | 615329877 | 27.03 | 100.23 | GMBA |
| *H. limprichtii* | GBIF | 1304083159 | 27.82308 | 99.706013 | KUN |
| *H. limprichtii* | GBIF | 2417629806 | 27.92 | 101.27 | PE |
| *H. limprichtii* | GBIF | 1304083190 | 27.928607 | 101.279419 | KUN |
| *H. limprichtii* | GBIF | 1030869900 | 28.932073 | 99.79929 | CDBI |
| *H. limprichtii* | GBIF | 1030890552 | 29.000343 | 101.507299 | CDBI |
| *H. limprichtii* | GBIF | 1030869897 | 29.037008 | 100.298406 | CDBI |
| *H. limprichtii* | GBIF | 2417755842 | 29.22 | 102.35 | PE |
| *H. davidii* | GBIF | 919594887 | 23.363 | 104.250727 | PE |
| *H. davidii* | GBIF | 1304083290 | 24.580446 | 99.92854 | KUN |
| *H. davidii* | Our own data (LWS) |  | 24.75541389 | 102.8714194 | KUN |
| *H. davidii* | GBIF | 1999305248 | 25 | 100 | A |
| *H. davidii* | GBIF | 919554072 | 25.012526 | 102.700013 | PE |
| *H. davidii* | Our own data (KM) |  | 25.19213611 | 102.7964389 | KUN |
| *H. davidii* | GBIF | 147033315 | 25.5 | 100.183 | A |
| *H. davidii* | GBIF | 1304083272 | 26.11116 | 99.951049 | KUN |
| *H. davidii* | GBIF | 1303742170 | 26.679722 | 106.33697 | PE |
| *H. davidii* | GBIF | 1304083276 | 26.876096 | 100.229945 | KUN |
| *H. davidii* | GBIF | 2417461322 | 26.91 | 104.2 | PE |
| *H. davidii* | GBIF | 1998941515 | 27 | 100 | GH |
| *H. davidii* | GBIF | 2417612949 | 27.03 | 108.19 | PE |
| *H. davidii* | This study (DB) |  | 27.03802222 | 100.2595056 | KUN |
| *H. davidii* | GBIF | 1304083293 | 27.177166 | 99.28717 | KUN |
| *H. davidii* | GBIF | 2417546468 | 27.71 | 102.8 | PE |
| *H. davidii* | GBIF | 1030889466 | 27.711428 | 102.807301 | CDBI |
| *H. davidii* | GBIF | 919554052 | 27.740736 | 98.66582 | PE |
| *H. davidii* | GBIF | 1304083252 | 27.82308 | 99.706013 | KUN |
| *H. davidii* | GBIF | 1030897717 | 27.928607 | 101.279419 | CDBI |
| *H. davidii* | GBIF | 1030889515 | 28.328636 | 103.132181 | CDBI |
| *H. davidii* | GBIF | 2417390725 | 28.85 | 85.29 | PE |
| *H. davidii* | GBIF | 2417749363 | 28.96 | 102.77 | PE |
| *H. davidii* | GBIF | 1030897700 | 28.966068 | 102.771746 | CDBI |
| *H. davidii* | GBIF | 2417684390 | 29 | 109.85 | PE |
| *H. davidii* | GBIF | 1030897732 | 29.037008 | 100.298406 | CDBI |
| *H. davidii* | GBIF | 2417527557 | 29.16 | 103.14 | PE |
| *H. davidii* | GBIF | 1303742197 | 29.351675 | 102.678073 | PE |
| *H. davidii* | GBIF | 2417699953 | 29.85 | 95.76 | PE |
| *H. davidii* | GBIF | 2417703570 | 29.91 | 102.23 | PE |
| *H. davidii* | GBIF | 1030897723 | 30.066708 | 102.758313 | CDBI |
| *H. davidii* | GBIF | 2575061377 | 30.7 | 102.7 | A |
| *H. davidii* | GBIF | 1303742171 | 30.996299 | 103.612264 | PE |
| *H. davidii* | GBIF | 2417691371 | 31.08 | 103.26 | PE |
| *H. davidii* | GBIF | 1030889430 | 31.084 | 103.267 | CDBI |
| *H. delavayi* | GBIF | 1030947703 | 22.99417 | 102.392353 | IBK |
| *H. delavayi* | GBIF | 1998613018 | 23.3 | 103.4 | AMES |
| *H. delavayi* | GBIF | 1303967066 | 24.35821 | 102.748636 | IBSC |
| *H. delavayi* | GBIF | 147033356 | 24.9333 | 102.483 | A |
| *H. delavayi* | GBIF | 1998825628 | 25 | 100 | A |
| *H. delavayi* | GBIF | 919554177 | 25.012526 | 102.700013 | PE |
| *H. delavayi* | Our own data (TS) |  | 25.25358889 | 102.7201611 | KUN |
| *H. delavayi* | GBIF | 615329879 | 25.27 | 100.97 | GMBA |
| *H. delavayi* | GBIF | 1304083287 | 25.584234 | 100.222124 | KUN |
| *H. delavayi* | GBIF | 147033303 | 25.6333 | 100.167 | A |
| *H. delavayi* | GBIF | 919554170 | 25.869611 | 102.593465 | PE |
| *H. delavayi* | GBIF | 1999304024 | 26 | 100 | GH |
| *H. delavayi* | GBIF | 1304083230 | 26.11116 | 99.951049 | KUN |
| *H. delavayi* | GBIF | 1030897712 | 26.634503 | 102.577962 | CDBI |
| *H. delavayi* | GBIF | 1304083241 | 26.876096 | 100.229945 | KUN |
| *H. delavayi* | This study (DB) |  | 26.99059722 | 100.2042306 | KUN |
| *H. delavayi* | GBIF | 919554166 | 27.740736 | 98.66582 | PE |
| *H. delavayi* | GBIF | 1303742154 | 27.928607 | 101.279419 | PE |
| *H. delavayi* | GBIF | 2417351214 | 28.54 | 102.17 | PE |
| *H. delavayi* | GBIF | 147033531 | 28.7433 | 101.681 | A |
| *H. delavayi* | GBIF | 1228375368 | 28.7433 | 101.6808 | F |
| *H. delavayi* | GBIF | 1303742162 | 29.000343 | 101.507299 | PE |
| *H. delavayi* | GBIF | 1030897720 | 29.037008 | 100.298406 | CDBI |
| *H. delavayi* | GBIF | 2417758146 | 29.22 | 102.35 | PE |
| *H. delavayi* | GBIF | 2417705610 | 29.91 | 102.23 | PE |
| *H. delavayi* | GBIF | 1030870428 | 29.914155 | 102.234618 | CDBI |
| *H. delavayi* | GBIF | 1057553919 | 31.712092 | 88.118532 | NHMUK |
| *H. delavayi* | GBIF | 3042972465 | 35.86 | 104.2 | KUN |
| *H. delavayi* | GBIF | 1055303706 | 36.519977 | 103.891767 | NHMUK |

**Table S2.** The flowering phenology of three *Habenaria* species from different sites in 2017. Flowering plants of *H. limprichtii* (n = 30), *H. davidii* (n = 27), *H. delavayi* (n = 34) in each site were monitored and recorded.

| **Study species** | **Study sites** | | |
| --- | --- | --- | --- |
| *H. limprichtii* | YSZ | DBWG | JLZ |
|  | Aug. 3rd - Sept. 16th | Aug. 19th - Sept. 14th | Aug. 5th - Sept. 15th |
| *H. davidii* | JQMK | CSZC | BSZT |
|  | Aug. 3rd - Sept. 15th | Aug. 7th - Sept.17th | Aug. 20th - Oct. 2nd |
| *H. delavayi* | YSZ | DBWG | JLZ |
|  | Jul.19th - Aug. 20th | Jul. 22 - Aug. 25 | Jul. 21th - Aug. 23th |

**Table S3.** The plant morphology of three *Habenaria* species in four populations on Yulong Snow Mountain, Lijiang, northwest Yunnan. For the data of plant morphology, *H. limprichtii* (data from YSZ, n = 70), *H. davidii* (data from JQMK, n = 79), *H. delavayi* (data from YSZ and DBWG, n = 57 (left), 80 (right)). Values followed by different letters on the same line differ significantly (P < 0.001). For one population (YSZ) of *H. delavayi*, we did not measure “distance between the two viscidia”, and denoted by NA. In principal coordinates analysis (PCoA), we replaced the value of the distance between two viscidia in this population (n = 57) with the mean (0.76) of this trait of the in population (JQMK).

| **Traits** | ***H. limprichtii*** | ***H. davidii*** | ***H. delavayi*** | |
| --- | --- | --- | --- | --- |
| **Length of lateral petal (mm)** | 18.87 ± 1.32 ^a^ | 17.68 ± 1.59 ^b^ | 7.27 ± 1.16 ^c^ | 7.77 ± 0.95 ^c^ |
| **Width of lateral petal (mm)** | 5.26 ± 0.69 ^a^ | 4.04 ± 0.53 ^b^ | 1.03 ± 0.20 ^c^ | 1.08 ± 0.16 ^c^ |
| **Spur length (mm)** | 20.33 ± 1.62 ^a^ | 32.87 ± 3.31 ^b^ | 18.22 ± 1.70 ^c^ | 18.93 ± 1.52 ^c^ |
| **Width of spur opening (mm)** | 2.32 ± 0.38 ^a^ | 1.38 ± 0.25^b^ | 1.27 ± 0.25 ^b^ | 1.34 ± 0.21 ^b^ |
| **Length of flower (mm)** | 43.83 ± 3.45 ^a^ | 47.00 ± 3.76 ^b^ | 18.11 ± 2.14^c^ | 19.42 ± 1.67^c^ |
| **Distance between two pollinium (mm)** | 9.50 ± 0.85 ^a^ | 3.53 ± 0.53 ^b^ | 1.42 ± 0.28^c^ | 1.52 ± 0.22 ^c^ |
| **Distance between the two stigma lobes (mm)** | 2.62 ± 1.10^a^ | 2.12 ± 0.93^b^ | 0.29 ± 0.14^c^ | 0.30 ± 0.12 ^c^ |
| **Distance between the two viscidia (mm)** | 4.25 ± 0.91 ^a^ | 3.64 ± 0.71 ^b^ | NA | 0.76 ± 0.14 ^c^ |
| **Length of lateral sepal (mm)** | 19.42 ± 1.43 ^a^ | 18.02 ± 1.68 ^a^ | 5.95 ± 1.11 ^b^ | 5.39 ± 0.73 ^b^ |
| **Width of lateral sepal (mm)** | 19.42 ± 1.43 ^a^ | 18.02 ± 1.68 ^b^ | 5.95 ± 1.11 ^c^ | 5.39 ± 0.73 ^c^ |

**Table S4.** The loadings of the principal coordinate axes. The percentage of variance explained by the different components was listed in brackets after each component.

|  | **Comp.1 (89.66)** | **Comp.2 (8.67)** | **Comp.3 (0.66)** | **Comp.4 (0.28)** | **Comp.5 (0.26** | **Comp.6 (0.17)** | **Comp.7 (0.13)** | **Comp.8 (0.08)** | **Comp.9 (0.06)** | **Comp.10 (0.02)** | |
| --- | --- | --- | --- | --- | --- | --- | --- | --- | --- | --- | --- |
| **Length of lateral petal** | 0.310 | 0.168 | 0.274 | 0.272 | -0.830 | -0.180 |  |  |  |  |  |
| **Width of lateral petal** |  | 0.110 |  | 0.102 | 0.128 |  |  |  | 0.969 |  | |
| **Spur length** | 0.272 | -0.840 | 0.366 | 0.248 | 0.120 |  |  |  |  |  | |
| **Width of spur opening** |  |  |  |  |  |  |  |  |  | -0.988 | |
| **Length of flower** | 0.790 |  | -0.600 |  |  |  |  |  |  |  | |
| **Distance between two pollinium** | 0.129 | 0.424 | 0.241 | 0.580 | 0.321 | 0.188 | -0.417 | 0.207 | -0.182 | 0.145 | |
| **Distance between the two stigma lobes** |  |  |  |  | 0.218 | -0.893 |  | 0.381 |  |  | |
| **Distance between the two viscidia** |  |  | 0.105 | 0.116 | 0.195 | -0.325 | -0.117 | -0.896 |  |  | |
| **Length of lateral sepal** | 0.376 | 0.209 | 0.579 | -0.661 | 0.120 | 0.133 |  |  |  |  | |
| **Width of lateral sepal** | 0.162 | 0.136 | 0.128 | 0.232 | 0.271 |  | 0.885 |  | -0.145 |  | |

**Table S5.** The number of pollen germinating, the number of pollen tubes (PT) entering into styles and ovaries, and the proportion of pollen tubes entering into styles and ovaries of three *Habenaria* species following intra- and interspecific cross-pollination. *lim* = *H. limprichtii*, *dav* = *H. davidii*, *del* = *H. delavayi*.

| **Species** | **Entry stage of pollen tubes** | ***lim*♂** | ***dav*♂** | ***del*♂** |
| --- | --- | --- | --- | --- |
| *lim*♀ | No. pollen germinated on stigma | 847.25 ± 219.19 （N *=* 28） | 969.10 ± 287.39（N *=* 30） | 346.32 ± 125.28 （N *=* 31） |
|  | No. PTs entry into style | 722.68 ± 176.65 （N *=* 28） | 808.77 ± 245.82 （N *=* 30） | 76.77 ± 106.39 （N *=* 31） |
|  | No. PTs entry into ovary | 645.96 ± 170.00 （N *=* 28） | 723.83 ± 227.62 （N *=* 30） | 17.16 ± 61.60 （N *=* 31） |
|  | No. PTs entry into style / pollen germinated on stigma | 0.86 ± 0.12 （N *=* 28） | 0.84 ± 0.12 （N *=* 30） | 0.20 ± 0.18 （N *=* 31） |
|  | No. PTs entry into ovary / PTs entry into style | 0.89 ± 0.10 （N *=* 28） | 0.90 ± 0.08 （N *=* 30） | 0.10 ± 0.21 （N *=* 31） |
| *dav*♀ | No. pollen germinated on stigma | 1281.28 ± 412.93 （N *=* 29） | 697.48 ± 294.47 （N *=* 27） | 414.87 ± 204.10 （N *=* 32） |
|  | No. PTs entry into style | 1054.86 ± 282.57 （N *=* 29） | 577.26 ± 224.20 （N *=* 27） | 118.167 ± 59.908 （N *=* 32） |
|  | No. PTs entry into ovary | 867.00 ± 229.70 （N *=* 29） | 500.89 ± 190.72 （N *=* 27） | 4.10 ± 11.217 （N *=* 32） |
|  | No. PTs entry into style / pollen germinated on stigma | 0.85 ± 0.18 （N *=* 29） | 0.85 ± 0.11 （N *=* 27） | 0.32 ± 0.17 （N *=* 32） |
|  | No. PTs entry into ovary / PTs entry into style | 0.83 ± 0.12 （N *=* 29） | 0.88 ± 0.10 （N *=* 27） | 0.04 ± 0.09 （N *=* 32） |
| *del*♀ | No. pollen germinated on stigma | 224.25 ± 112.59 （N *=* 32） | 107.28 ± 98.79 （N *=* 29） | 499.65 ± 168.86 （N *=* 31） |
|  | No. PTs entry into style | 106.66 ± 109.10 （N = 32） | 41.07 ± 63.31 （N = 29） | 440.81 ± 156.97 （N = 31） |
|  | No. PTs entry into ovary | 44.41 ± 67.69 （N = 32） | 17.07 ± 37.84 （N = 29） | 380.87 ± 146.45 （N = 31） |
|  | No. PTs entry into style / pollen germinated on stigma | 0.41 ± 0.35 （N = 32） | 0.21 ± 0.28 （N = 29） | 0.88 ± 0.11 （N = 31） |
|  | No. PTs entry into ovary / PTs entry into style | 0.29 ± 0.30 （N = 32） | 0.33 ± 0.33（N = 29） | 0.87 ± 0.13（N = 31） |

**Table S6.** Fruit set and the proportion of seeds with large embryos in the three *Habenaria* species (*lim* = *H. limprichtii*, *dav* = *H. davidii*, *del* = *H. delavayi*) resulting from controlled intra- and interspecific crosses.

| **Female parent** | **Male parent** | **No. plants** | **No. flowers** | **No. fruits** | **Fruit set (%)** | **Large embryo rate** |
| --- | --- | --- | --- | --- | --- | --- |
| *lim*♀ | *lim*♂ | 45 | 88 | 86 | 97.72 | 0.71 ± 0.19（N = 30） |
|  | *dav*♂ | 38 | 65 | 64 | 98.46 | 0.65 ± 0.18（N = 64） |
|  | *del*♂ | 35 | 51 | 0 | 0 | 0.00 ± 0.00（N = 51） |
| *dav*♀ | *dav*♂ | 40 | 79 | 74 | 93.67 | 0.75 ± 0.22（N = 41） |
|  | *lim*♂ | 31 | 59 | 55 | 93.22 | 0.77 ± 0.16（N = 55） |
|  | *del*♂ | 32 | 60 | 0 | 0 | 0.00 ± 0.00（N = 60） |
| *del*♀ | *del*♂ | 31 | 50 | 49 | 98.00 | 0.66 ± 0.16（N = 46） |
|  | *lim*♂ | 33 | 53 | 0 | 0 | 0.00 ± 0.00（N = 53） |
|  | *dav*♂ | 36 | 59 | 0 | 0 | 0.00 ± 0.00（N = 59） |
